# Biometals in Alzheimer disease: emerging therapeutic and diagnostic potential of molybdenum and iodine

**DOI:** 10.1186/s12967-023-04220-5

**Published:** 2023-05-27

**Authors:** Benson O. A. Botchway, Xuehong Liu, Yu Zhou, Marong Fang

**Affiliations:** 1 Department of Neurology, Children’s Hospital of Zhejiang University School of Medicine, National Clinical Research Centre for Child Health, Hangzhou, 310052 China; 2 Pharmacy Department, Bupa Cromwell Hospital, Kensington, London, SW5 0TU UK; 3 Department of Histology and Embryology, Medical College, Shaoxing University, Shaoxing, Zhejiang China

**Keywords:** Neurodegeneration, Trace metal dyshomeostasis, Metallobiology

## Abstract

The current ageing trend of the world population has, in part, accounted for Alzheimer disease (AD) being a public health issue in recent times. Although some progress has been made in clarifying AD-related pathophysiological mechanisms, effective intervention is still elusive. Biometals are indispensable to normal physiological functions of the human body—for example, neurogenesis and metabolism. However, their association with AD remains highly controversial. Copper (Cu) and zinc (Zn) are biometals that have been investigated at great length in relation to neurodegeneration, whereas less attention has been afforded to other trace biometals, such as molybdenum (Mo), and iodine. Given the above context, we reviewed the limited number of studies that have evidenced various effects following the usage of these two biometals in different investigative models of AD. Revisiting these biometals via thorough investigations, along with their biological mechanisms may present a solid foundation for not only the development of effective interventions, but also as diagnostic agents for AD.

## Background

In the last five decades, technological inventions and enhancements have attempted to improve our understanding of AD. β-amyloid accumulation, along with several determinants like tau phosphorylation, oxidative stress, dyshomeostases of the gut microbiome and biometals have been linked to AD neuropathology. In our previous report, some of these factors were thoroughly analyzed [1]. Despite unclear mechanisms, some of these determinants appear to work both synchronously and independently. For example, oxidative stress augments β-amyloid levels to cause neurodegeneration. Correspondingly, accumulated β-amyloid triggers mitochondrial dysfunction, leading to oxidative stress [2]. Therefore, to target only one factor of the disease may not effectively result in a significant improvement. Similarly, as AD has got an intricate pathophysiological mechanism, it is possible that a combination of different interventional agents may have to be employed to effectively manage and treat the disease. In recent times, interventional agents (sodium oligomannate, aducanumab and lecanemab) have been approved by health agencies to manage the condition [2–4]. The more recent approval of aducanumab has received a lot of criticisms from research and medical experts, with Walsh and colleagues doing a comprehensive thought-provoking editorial in the BMJ [5]. Most of the criticisms are in relation to the lack of substantial evidence to necessitate its approval. Indeed, aducanumab does significantly mitigate β-amyloid levels. However, whether decreased β-amyloid by aducanumab is concomitant with improved cognition and ADCS-ADL (Alzheimer's Disease Co-operative Study-Activities of Daily Living Inventory) to justify its approval for usage in the clinical setting is unclear. Although one clinical trial showed aducanumab to meet both its primary and secondary clinical objectives (EMERGE) amid longer follow-up and increased dose, results from another clinal study (ENGAGE) showed the contrary [6, 7].

Trace biometals like Cu and Zn have been heavily investigated in relation to AD [8]. Both Cu and Zn are key players in oxidative stress, protein misfolding and aggregation [9, 10]. Although these biometals have been investigated at great length, minimal progress has been made in terms of their utilization as interventional agents or targets for AD. The excessive research has presented several conflicting reports to the extent of some researchers calling for the discontinuation of metal chelators for AD, while others are still in favor of them [11, 12]. Further, the tremendous studying of Cu and Zn has led to less attention being afforded to other trace biometals that are used by the human body. Cobalt is a trace biometal and a major factor in vitamin B_12_ synthesis. This vitamin necessitates several neurological functions including cognition [13]. There are reports showing that decreased serum vitamin B12 may enhance neurodegenerative disease risk [14, 15]. Until recently, no study had reported the connection between cobalt and PIN-1 (Peptidyl-prolyl cis–trans isomerase NIMA-interacting 1) in neurodegeneration. PIN-1 has been demonstrated to cause AD when its expression is downregulated [16]. Contrastingly, when its expression is upregulated, cancer may be triggered [16, 17]. In a recent study, cobalt decreased PIN-1 expression, and halted the G_0_/G_1_ phase of the cell cycle by curtailing cyclin D protein levels, which in turn resulted in apoptosis of H4 human neuroglioma cells. Furthermore, in increasing the concentration of cobalt, disrupted activity and function of PIN-1 mice were discerned. In the in-vivo analysis using C57BL/6J mice, significant levels of cobalt were detected in the hippocampus, cortex, and blood. This coincided with mitigated levels of PIN-1, culminating in aggravated phosphorylated tau protein, β-amyloid protein, cognitive dysfunction, and neuronal loss in both hippocampus and cortex. More importantly, the same study analyzed blood samples of patients who had undergone metal-on-metal hip replacements. Following assessment, increased amount of cobalt was observed and was concomitant with reduced PIN-1 protein [18]. Although the study results are interesting, it is also a cause for concern. This is because patients who have had such hip replacements might need regular blood checks to ascertain the level of cobalt and modulate its level should there be an increase beyond the safety threshold, thereby preventing the potential development of AD.

**Fig. 1:**
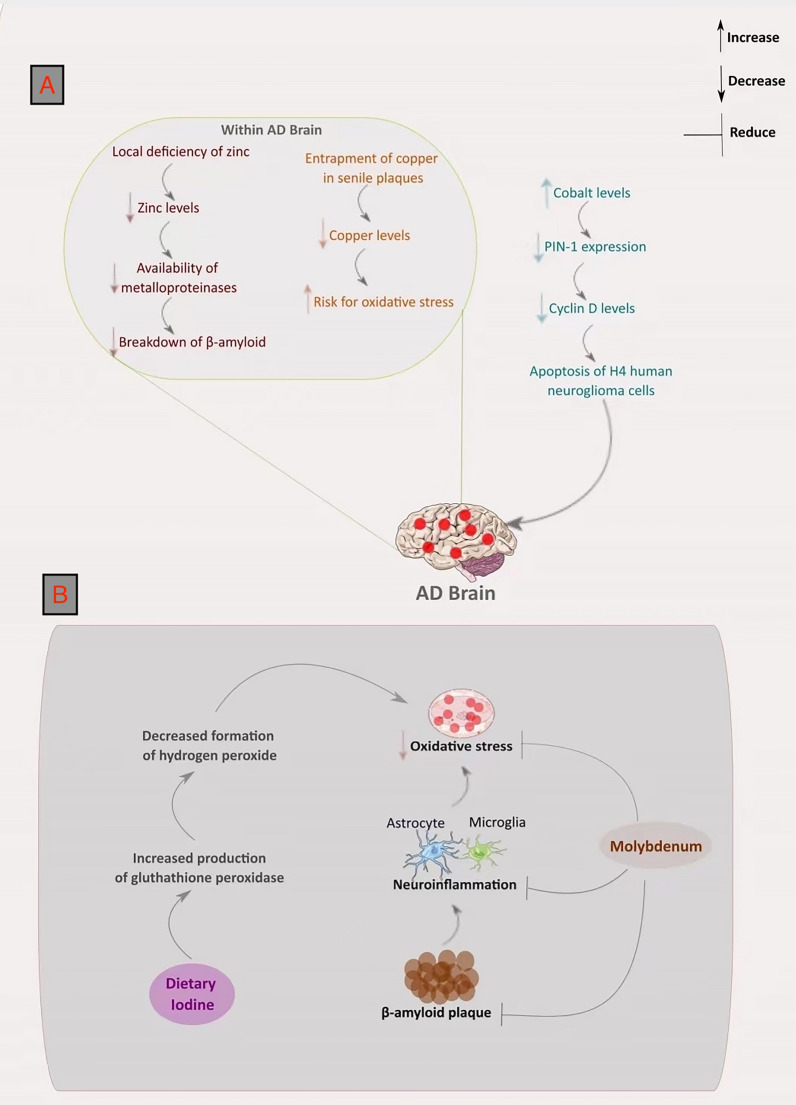
**a** The effects of zinc, copper, and cobalt deficiencies in AD. In AD brain, the paucity of zinc limits the accessibility of metalloproteinase, and causes β-amyloid to aggregate. The built-up β-amyloid that forms plaques traps copper and abate its level, subsequently expediting the possibility of oxidative stress. Similarly, the AD brain has augmented levels of cobalt, which potentially downregulates PIN-1 expression and decreases the level of cyclin D. Downregulated PIN-1 expression instigates cognitive dysfunction by accelerating phosphorylated tau protein and β-amyloid accumulation. **b** The therapeutic effect of molybdenum and iodine. Dietary iodine may counteract oxidative stress in AD by mitigating hydrogen peroxidation formation and enhancing the output of glutathione peroxidase. Similarly, molybdenum may impair neuroinflammation through the inhibition of astrocyte and microglia formation, and result in hindering both oxidative stress and β-amyloid

**Fig. 2:**
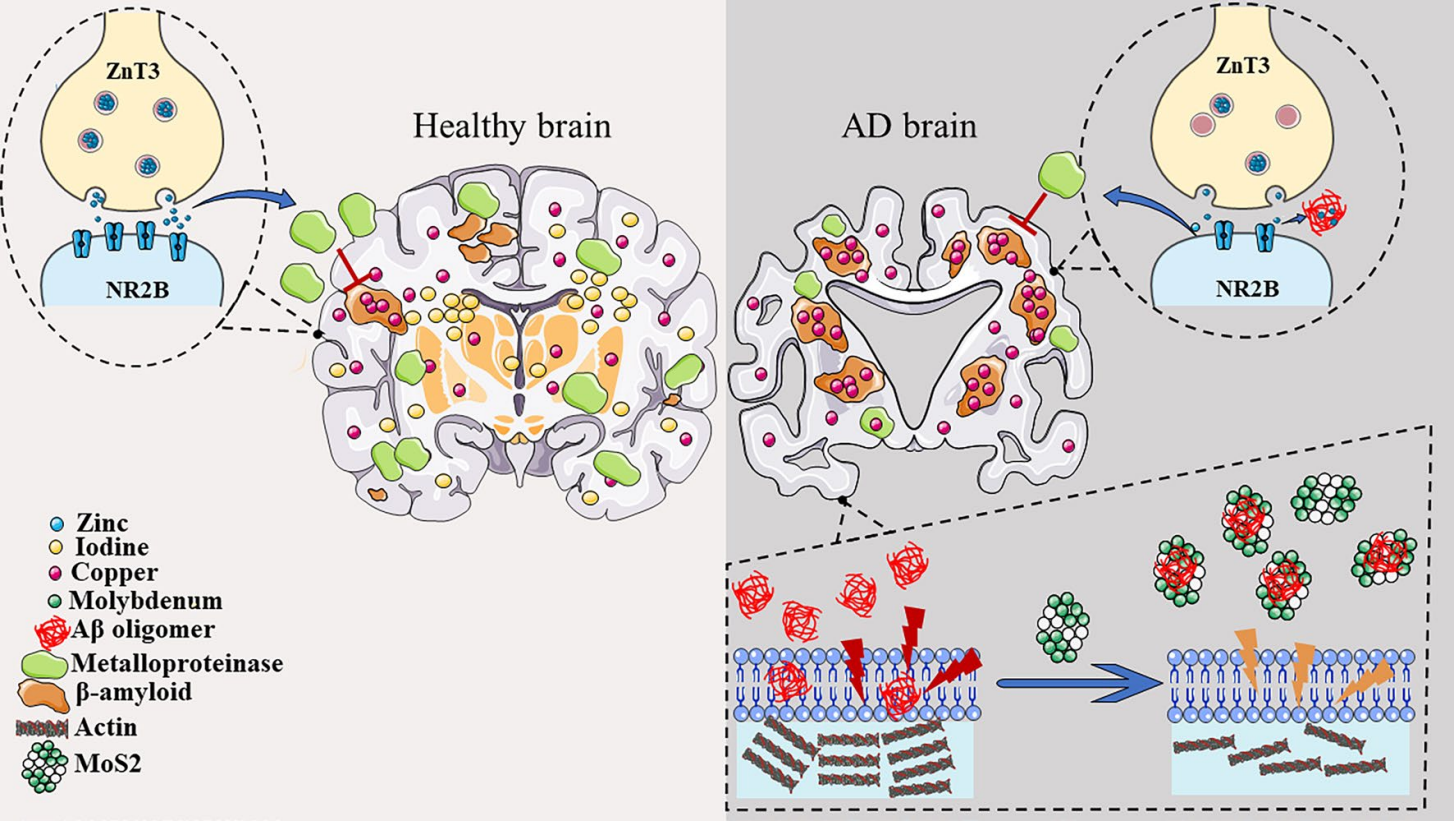
The different levels of zinc, copper, molybdenum, and iodine between healthy brain and AD brain. Metalloproteinases can break down β-amyloid, with zinc necessitating that function. Zinc transporters are abnormally expressed in AD brains. As zinc is displaced in AD, metalloproteinases are reduced, with the resultant effect of accumulated β-amyloid protein in the brain. Whereas some brain regions in AD patients might have lower copper levels, other areas may be in excess. Furthermore, AD brains (specifically, within the β-amyloid plaques) have higher concentration of copper than healthy brains. Besides, β-amyloid oligomers instigate cell membrane disruption and alter actin structure. However, the usage of MoS_2_ quantum dots may reverse these detrimental effects. Concerning iodine, its level in the brain of healthy individuals may be low and unevenly distributed. Notwithstanding, brain regions involved in cognition appear to have highest level of iodine

The study above underlies the need for thorough investigation of other trace biometals in AD. In that regard, we analyzed the role of two biometals (Mo and iodine) that we believe that been largely overlooked. We elaborate on their potentiality in not only as prospective interventional agents, but also as diagnostic medium for the disease. Lastly, we present figures that summarizes our report. Figure 1a shows the effects of biometal deficiencies leading to AD, Fig. 1b illustrates the potential effectiveness of molybdenum and iodine in countering AD, and Fig. 2 differentiates the level of Zn, Cu, molybdenum (Mo), and iodine between a healthy brain and an AD brain.

### Overview of copper and zinc in AD

Considering the extensive reportage, we summarize some studies that have attempted to elucidate the link regarding Zn and Cu in AD.

#### Copper (Cu)

Cu is a key trace element important for all oxygen-requiring processes, neurotransmitter synthesis, and neuronal myelination. In addition, Cu regulates the brain’s metabolic needs owing to its involvement in energy and iron metabolism [19–21]. Cu, as an essential cofactor, readily binds to enzymes and shifts between the Cu^2+^ and Cu^+^ oxidative states [19]. The brain is susceptible to oxidative stress triggered by the redox nature of Cu given that about 7.3% of total body Cu content is present in the brain [19]. Cu homeostasis is tightly regulated and mediated through trafficking and transportation. Ceruloplasmin is one of the main Cu-binding proteins in the plasma. In both serum and brain of AD patients, ceruloplasmin levels are elevated [22, 23]. Besides, cultured primary and secondary microglia have been used to evidence the instigation of proinflammatory response by ceruloplasmin. The inflammatory response was demonstrated by enhanced NO release and cytokines, such as tumour necrosis factor (TNF) and interleukin-1 beta (IL-1β) [24].

Cu, as an essential neuro-regulator, is released into the synaptic cleft of glutamatergic synapses during neurotransmission at micromolar concentrations [19, 25]. Free ionic Cu released at NMDA-responsive synapses activates the NMDA receptor. It is therefore not surprising that glutamatergic dysfunction in AD brain may be partly due to Cu dyshomeostasis [26]. Initially, Cu was thought to be a negative regulator of neurotransmission. However, a recent finding showed Cu to have a unique biphasic mechanism in neurotransmission [27]. In that study, hippocampal neurons of Sprague–Dawley rats exposed to Cu for 3 h resulted in augmented AMPAergic neurotransmission. This, in part, might have been due to the accumulated AMPA receptors at the plasma membrane [27].β-amyloid peptides are generated through amyloid precursor protein (APP) proteolysis [28]. APP is a transmembrane glycoprotein involved in axonal transport, vesicular trafficking, and neuronal survival. There are two alternate pathways of APP processing: the amyloidogenic and nonamyloidogenic pathways [29, 30]. The APP has two Cu-binding binding domains, one localized in the β-amyloid region and the other in the N-terminus. The presence of Cu (II) reductase activity within the Cu-binding domain of APP may potentiate ROS formation via Cu reduction [19]. Regarding the amyloidogenic processing of the APP, β-amyloid is formed through the cleavage of APP by β- and γ-secretases [29, 30]. In the nonamyloidogenic pathway, APP generates p3 peptide after it undergoes cleavage by α- and γ-secretases. Promotion of the amyloidogenic pathway and increased synthesis of β-amyloid have been consistently linked to AD neuropathology.

It is worth noting that modulation of secretases’ expression by metal ions is most likely to play a role in determining the pathway by which the APP is processed. Zn has been implicated in the regulation of α-secretase and γ-secretase activities [31, 32]. The interaction between Cu and β-secretase BACE 1 promotes the amyloidogenic processing of the APP. Moreover, Cu modifies the APP not only through the Cu-binding domain but also its processing and control of β-amyloid synthesis [33]. For instance, the work by Cater and colleagues showed that altered levels of intracellular Cu may influence the APP metabolism. In addition, elevated intracellular Cu enhanced the secretion of the α-cleaved APP, while the β-cleaved APP formation and secretion were higher in Cu-deficient cells [34].

In AD patients, some brain regions appear to have excess Cu while other areas are deficient. This mis-localization of Cu has significant effects on neuronal function, having been implicated in cognitive deficits and other AD neuropathological facets [35, 36]. In affected areas of AD brains, Cu levels are markedly curtailed and could be attributed to its entrapment in senile plaques. Specifically, the Cu content in β-amyloid plaques was nearly five-fold higher than normal age-matched controls. Additionally, tissues surrounding the senile plaques showed lowered Cu levels, indicating a possible local Cu deficiency [37].

Post-mortem examination remains the best option in ascertaining the amount of Cu in the brain and to directly detect β-amyloid plaques [35]. Measuring serum Cu may provide an insight regarding the extent of Cu in living patients, and thus, could be a prospective diagnostic tool for AD [38]. Noteworthy is that serum Cu can be in the form of non-ceruloplasmin-bound-copper (non-Cp–Cu) or bound to either ceruloplasmin or albumin. It is the uptake of free Cu ion that passes both the blood–brain barrier (BBB) and blood-cerebrospinal fluid barrier and is distributed to the CSF and brain parenchyma. In comparison to non-healthy controls, AD patients have higher copper serum levels (particularly non-Cp–Cu) [38–40].

In summary, Cu is necessary for various neurological functions—neuronal myelination, neurotransmitter formation and brain metabolism. Presently, there is significant gaps regarding the specific level of Cu in AD. While senile plaques may partly account for Cu paucity in some sections of the brain, what could be the rationale behind its augmented serum content in AD? What will be the best strategy in shifting Cu in the serum to other Cu-deficient compartments? In any case, will the effectiveness of that strategy correspond with significant improvement in AD? Future studies addressing some of these uncertainties could be instrumental in the development of an effective interventional mechanism for AD.

#### Zinc (Zn)

Zn is an essential trace biometal that maintains the function of various metalloenzymes in diverse non-enzymatic biological reactions and serves as a crucial component in hormone-receptor interactions, neurotransmission, and neurogenesis [41, 42]. It participates in signal transduction processes. As a neuro-regulator, Zn modulates brain excitability by inhibiting excitatory and inhibitory receptors. Noteworthy is that Zn qualifies as both neurotransmitter and second messenger [43]. It regulates synaptic plasticity in the form of long-term potentiation (LTP) and long term-term depression (LTD), which underlies learning and memory. Both LTP and LTD are regulated by the NMDA (*N*-methyl-d-aspartate) receptor subunits. Far more, Zn is co-released with glutamate into the synaptic cleft to control the activity of post-synaptic proteins, NMDA and AMPA receptors (α-amino-3-hydroxyl-5-methyl-4-isoxazolepropionate-acid) [43–45].

Zn homeostasis in the brain is primarily modulated by metallothioneins, Zn transporters, and members of the ZiP (zinc-regulated and iron-regulated transporter proteins) family [46]. Metallothionein (MT) is a Zn and Cu modulator that induces antioxidant reaction. Growth inhibitory factor (i.e., MT-3), an isoform of metallothionein, is abundant in astrocytes, cerebellar cortex, and Zn-enriched neurons. In AD brain, MT-3 level is considerably lower. Reduced MT-3 and loss of its protective effects may exacerbate AD pathogenesis. Besides, MT-3 has been associated with various neurodegenerative diseases, such as amyotrophic lateral sclerosis, Parkinson disease, and prion disease [47, 48]. Zn transporters are divided into two major families: SLC_30_ (ZnTs1-10) and SLC_39_ (ZiPs1-14). In the cytoplasm, SLC_30_ and SLC_39_ families of zinc transporters decreases and increases intracellular Zn level respectively [49]. ZnT1 is ubiquitously expressed and exports Zn to the extracellular space of the brain. Its interaction with the GluN2A-containing NMDA receptors forms the GluN2A/ZnT1 complexes and modulates postsynaptic receptors [50]. ZnT4 is present in lysosomal and endosteum compartments of the hippocampus, and functions by loading cytoplasmic Zn in the secretory vesicles [51]. Also, ZnT6 is found in the membrane of the Golgi apparatus where Zn binds to the APP and inhibits the cleavage of the APP at the α-secretase site [52, 53]. Like ZnT1, the ZiP1 transporter is ubiquitously expressed in human tissues [54]. It increases with advancing age of the human frontal cortex [55]. In AD brains, ZiP1 level is markedly increased with disease progression and Braak staging [56]. Hence, it is possible that the upregulation of ZiP1 levels could be an attempt to maintain normal Zn homeostasis as cytoplasmic Zn concentration may decrease with AD progression [56]. Notwithstanding, further studies using appropriate animal models may provide some insight and improve our current understanding.

AD is classified into preclinical AD (PCAD), MCI, early-stage AD (EAD), and late-stage AD (LAD). PCAD patients have normal cognitive functions but with existing AD neuropathology, while MCI patients have memory loss and evidence of neuropathology but with normal daily activities [57, 58]. In AD, alteration of the SCL_30_ group of Zn transporter is dependent on the pathological phase of the disease. For instance, in PCAD patients, increased cytoplasmic Zn concentration is concomitant with downregulated and upregulated ZnT1 and ZnT6 expressions respectively in the hippocampus. In contrast, both EAD and LAD patients have increased expression levels of ZnT1, ZnT4, and ZnT6 [59, 60]. We are unsure of the specific reasons underlying these variations. Speculatively, during AD progression, increased level of ZnT1 might compel Zn accumulation in the extracellular space that in turn would aggravate β-amyloid deposition. In addition, Zn concentration in the trans-Golgi network increases due to upregulated ZnT6 expression that exacerbates β-amyloid aggregation through the inhibition of the APP cleavage by α-secretase. Conclusively, the effects of altered ZnT1 levels on NMDA receptor function, and how ZnT4 influences AD neurobiology is presently not clear and warrants further studies.

#### Controversies surrounding zinc in AD

Metalloproteinases are enzymes that can necessitate the breakdown of β-amyloid. Interestingly, Zn is required for the normal functioning of metalloproteinases [61]. In AD, Zn displacement curtails the overall availability of metalloproteinases within the brain. With the decrement of this enzyme, β-amyloid is likely to aggregate in the brain and gradually instigate AD-associated symptoms, such as cognitive decline. Therefore, attenuated metalloproteinases owing to Zn dysfunction could partly account for the possible rationale behind β-amyloid accumulation in the brain of AD patients. It is worth noting that the blood–brain barrier (BBB) limits the traversal of molecules and pathogens from the peripheral to the CNS, and Zn is no exception. Interestingly, when Zn^2+^ was conveyed across the BBB of APP23 mice, mitigated β-amyloid plaques, cytokines, and synaptic loss were observed at a significant degree [61].

Several contentions regarding Zn and its resultant effect in AD have been put forward. Notably, β-amyloid and APP proteins were exacerbated in APP/PS1 mice that were given water containing ZnSO_4_ (20 mg/mL or 20 ppm). This led to compromised memory and spatial learning [62]. In a subsequent preclinical study, hippocampal synaptic proteins (PSD—93&95, NR2B, NMDA-NR2A, AMPA-GluR1) and dendritic spines were considerably lower in 21-day old ICR mice that had been administered water containing high doses of Zn (60 ppm or 60 mg/mL). In addition, memory dysfunction, along with curtailed level of hippocampal BDNF and TrkB neurotrophic signaling were reported [63].

On the contrary, some reports have observed Zn supplementation to improve cognition and improve mitochondria function. For instance, Corona and colleagues did observe increased BDNF levels as well as decrement in both tau and β-amyloid pathologies in 3xTg-AD mice that had been administered with ZnSO_4_-supplemented tap water. The enhanced BDNF levels was related to the instigation of matrix metalloproteinases. Additionally, mitochondrial activities were restored in the hippocampal region [64]. In a more recent study, Zn supplementation improved the short- and long-term recognition memory of young rats as well as the short-term recognition memory and spatial working memory of adult rats. Interestingly, exacerbated Cu contents were neutralized by zinc supplementation [65].

Zn supplementation regulates oxidative stress. For example, adult male Wistar rats were exposed to cadmium for 6 months to induce oxidative stress in the brain. When Zn (either 30 or 60 mg/L) was administered to these animals, the cadmium-instigated oxidative damage was reversed. This was evidenced by the enhanced antioxidative markers (SOD, CAT, GPx) and mitigated pro-oxidant factors (such as myeloperoxidase and H_2_O_2_) [66]. In a meta-analysis study involving adults taking Zn supplements, decreased oxidative stress was observed following serum analysis. Interestingly, this also coincided with decreased inflammatory markers (TNF-α and C-reactive protein) [67].

While the precise role of Zn in neurodegeneration, particularly AD, remains a topic of interest, we believe its homeostasis in the brain must be tightly controlled. As such, studies addressing the optimal level of Zn in the brain necessary for favorable outcomes in AD would be worthwhile. Further, whether increase in Zn concentration in the brain via supplementation or diet has a direct effect on biomarkers such as SOD in curtailing oxidative stress in AD is presently unclear. In addition, whether excessive Zn level in the brain might compromise the function of SOD and lead to oxidative stress is open to question. To thoroughly understand Zn’s mechanism, feasibility, and potential applicability in AD, prospective studies will have to address some of these challenges.

### The need for investigations of other biometals: significance of molybdenum and iodine in AD

#### Iodine

Iodine is a biometal whose role in biological processes in humans cannot be overstated. Besides being involved in the metabolic processes of thyroid hormones (thyroxine (T4) and triiodothyronine (T3)), conditions such as goiter come about because of its decreased intake in diet [68, 69]. There is presently no study that has specifically investigated and correlated iodine levels directly to AD. However, several reports have attempted to link iodine and AD via thyroid hormones [70, 71]. Some of the association being made pertains to thyroid hormones involvement in neurotransmission, cognition, and hippocampal function, with reports such as that of Redman et al. thoroughly reviewing these relations [71]. As previously stated, synaptic plasticity promotes learning and memory. Therefore, its dysfunction is likely to affect cognition [72]. Although mechanism remains to be elucidated, thyroid hormones have been shown to affect synaptic plasticity in the hippocampal region [73]. Specifically, Gilbert and Sui used propylthiouracil to suppress thyroid hormone level in pregnant rats. This resulted in compromised spatial learning and LTP, concomitant with impaired synaptic plasticity [73].

Regarding neurotransmission, Smith and colleagues observed heightened acetylcholine activity in the frontal cortical and hippocampal sections of the brain when adult male rats were chronically administered l-thyroxine (5 mg/kg and 10 mg/kg). More importantly, this outcome coincided with improved cognitive performance [70]. Indeed, several clinical studies have been conducted in children to ascertain the association between cognition and iodine using various cognitive assessments, with positive correlation being observed [74–77]. Nonetheless, studies evidencing such association in AD patients is significantly lacking, at least to the best of our knowledge. Therefore, it remains to be seen what the outcome will be when similar investigations are replicated in AD patients.

#### Molybdenum

The physiological functions of Mo are numerous, from participating in the disintegration of toxic agents and drugs to being involved in genetic and protein processes [78]. In human biological processes, Mo functions as a co-factor that leads to the activation of enzymes like sulfite oxidase, aldehyde oxidase, xanthine oxidase, and mitochondrial amidoxime reducing component [79]. These enzymes are involved in various physiological functions, such as removal of toxic products and synthesis of uric acid that necessitate the degradation of nucleotides [79, 80]. Although rare, Mo deficiency causes detrimental health effects. In comparative studies that found low levels of Mo by analyzing nail and hair samples, there was a probable risk of esophageal cancer development in populates [81, 82]. Mo deficiency may indirectly be involved in AD. Sulfite oxidase instigates the conversion of sulfite to sulfate [83]. Increased sulfite levels, possibly through Mo deficiency, could compromise the human gut microbiota as recently observed [84, 85]. The human gut microbiota is still an evolving research area in AD. However, altered levels of some gut microbiota may cause AD as thoroughly reviewed from our previous study [1].

Xanthine oxidase catalyzes the formation of xanthine from hypoxanthine via oxidative mechanism, which is then converted to uric acid [79]. In comparison to age-matched controls, curtailed levels of xanthine and hypoxanthine were found in the frontal cortex of postmortem brain samples of AD individuals [86]. Mo’s involvement in the formation of uric acid is particularly interesting given that several investigations have attempted to evidence the relationship between serum uric acid and cognition. In several of these studies, there was a correlation between increased serum uric acid content and mitigated risk of developing AD and MCI, as well as improvement in cognitive decline [87–89]. The positive effects of uric acid in these studies, although interesting, is also a cause for concern as its (i.e., uric acid) augmented concentration causes gout [90]. Indeed, results from prospective cohort studies showed a lower risk of developing AD in patients with gout [91, 92].

Given the above studies, will it therefore be rational for individuals, especially those at high risk, to endure the symptoms of gout (such as joint pain and swelling) and mitigate their risk of developing MCI, AD, or slow cognitive decline? The answer is presently unclear; however, future studies may shed light on the right course of action. In view of the relationship between Mo and xanthine oxidase, it is possible that individuals residing in areas with low Mo levels or eating Mo-deficient diets may be at an increased risk of developing AD.

### Molybdenum: Could it be a prospective interventional agent for AD?

Mo has multiple functions in neurodegenerative diseases, especially in AD, from being a possible diagnostic agent to its ability to inhibit β-amyloid and regulate oxidative stress [93]. In the context of diagnostics for AD, Dou and colleagues engineered a two-dimensional assemblage of thin-layer molybdenum disulfate and quinoline-malononitrile aggregation induced emission. With its enhanced fluorescence features, the compound was better at detecting Aβ_42_ peptide accumulations in the brain of 12-month-old APP/PS1 transgenic mice when compared to the sole use of quinoline-malononitrile aggregation induced emission. The enhanced fluorescence feature of this flat assemblage was due to its effectiveness in permeating through the BBB [94]. Comparable studies such as that of Qu et al. also demonstrated the ability of a reaction of molybdate and hydroxyapatite to trigger an electrochemical current that can evaluate both functional and inhibitory activities of beta-site amyloid precursor protein cleaving enzyme-1 (BACE-1) [95]. We have previously showed BACE-1 to be regulated by miR-124 in AD, leading to alterations in autophagy expressions [96].

There is some evidence that Mo can counteract oxidative stress and β-amyloid. In several experimental models of AD, the antioxidant features of Mo-containing agents have been evidenced. β-amyloid triggers oxidative stress via the generation of ROS and are cytotoxic [1]. Nanoparticles of Mo oxide were synthesized using the pulsed laser ablation technique. Beside stimulating the clearance and hindering β-amyloid agglomeration, these nanoparticles also lowered ROS amount that had been triggered by β-amyloid [97]. CAT, just like SOD, is a potent antioxidant. The link between inflammation and oxidative stress has been reviewed [1], with animal models of AD also confirming the association [98]. Microglia M_1_ and M_2_ are inflammatory markers, with the M_1_ phenotype being proinflammatory while the M_2_ phenotype is anti-inflammatory. In a recent study, a constructed nanoparticle (molybdenum disulphide quantum dots and 1,2-distearoyl-sn-glycero-3-phosphoethanolamine-*N*-[amino (polyethylene glycol)-2000] that was aimed at the mitochondria was effective in not only penetrating the BBB, but also afforded protection to both neuronal cells and microglial against β-amyloid. Specifically, the in-vitro analysis using BV-2 cells showed the constructed nanoparticle to safeguard against β-amyloid-instigated mitochondria destruction, and moderate ROS level that had been triggered by β-amyloid, while significantly modulating neuroinflammation via corresponding downregulated and upregulated proinflammatory (TNF-α, interleukin (IL)-6, and IL-1β) and anti-inflammatory (TGF-β) expressions. In using APP/PS1 transgenic mice for the in-vivo assessment, a significant upregulated CD206 expression and downregulated CD16/32 expression were observed in the brain. In addition, there was marked mitigation of Iba-1 level and oxidative stress (via downregulated 4-hydroxynoneal) in the hippocampal region [99]. Two deductions are worth noting from the above study, and in both cases relate to the specificity of the nanoenzyme. In the brain of the transgenic mice used for the study, the constructed nanoenzyme was specifically targeting the hippocampus and not the cortices, as β-amyloid were noted to be lower in that section. Thus, is it possible that a nanoenzyme focusing on the mitochondria could be significantly beneficial to the hippocampus and have minimal or no improvement to the cortex? That remains to be clarified through additional studies using various disease models. The other intriguing aspect of the study had to do with the detection of this nanoenzyme in other organs (liver, kidney, spleen, and lung) to the extent of minimizing both oxidative and inflammatory damages in renal tubules, as well as not affecting the immunological activities of the spleen. In addition, the RT-qPCR analysis of both TGF-β and TNF-α genes showed respective increase and decrease, together with no disruptions to the functions of antioxidant enzymes (CAT and SOD) in the lung of transgenic mice. Conclusively, detrimental effects were not reported in these organs, indicating that not only is the nanoenzyme effective, but also possess no risk to other areas of the body. Contrastingly, other studies have reported detrimental effects, such as intestinal damage, downregulated antioxidant enzyme gene expression, apoptosis, and ROS production following the usage of molybdenum disulphide (MoS_2_) [100, 101]. The difference between these two studies [100, 101] and that of Ren et al. [99] was the absence of complexation. Therefore, it is plausible the presence of a potent molybdenum complexation could have mitigated these unfavorable effects, as has been observed [102]. Nonetheless, well-organized future preclinical studies may clarify these contradictions.

More recent analysis using Mo-containing agents in different investigative models of AD have been reported. Notably, Sudipa et al. employed insulin protein as an in-vitro method and found ammonium molybdate to significantly suppress generated β-amyloid fibrils. Their result was substantiated via the in-vivo route using drosophila fly. Interestingly, no harmful effects were reported [103]. A similar study by Linga and colleagues attained similar results. Their study utilized nanosheets of MoS_2_ to successfully detect β-amyloid oligomers and significantly suppress its aggregation [104].

Actin, as a protein of multi-functional value, is involved in microfilament formation and takes a role in the modulation of DNA replication and cell motility [105, 106]. The potential association between dysregulated actin and AD has been extensively investigated. Specifically, the correlation between AD and actin appears to be through dysfunctionality in gelsolin and cofilin-1 proteins (i.e., actin-binding proteins) [107–109]. For instance, recent investigations showed β-amyloid oligomers to trigger the phosphorylation of cofilin-1 protein, causing its augmentation in both APP/PS1 mice and AD patients, and leading to curtailed synaptic density and plasticity. More importantly, when fasudil was used to limit the ROCK pathway (Rho-associated protein kinase), the stimulated effects were abrogated [110]. In the study by Li et al., they observed β-amyloid oligomers to instigate disruption to the cell membrane and alter the actin structure. Nevertheless, the application of MoS_2_ quantum dots (in SH-SY5Y cells) counteracted these detrimental effects, resulting in reduced oxidative stress and cell death [111]. The only limitation to this study had to do with being an in-vitro analysis. Therefore, it would be interesting if similar results could be achieved following its replication in different animal models of AD, while also examining other AD pathological features, such as neurofibrillary tangles (NFT), synaptic plasticity and density, and cognition. More importantly, the delineation of the mechanisms involved could be significant in facilitating the employment of this nanoparticle as an interventional agent for AD. Table 1 summarizes other fabricated nanoparticles containing Mo that have shown potentiality in improving some of the neuropathological facets of AD.

**Table 1:** Summary of fabricated nanoparticles containing molybdenum employed for AD in preclinical studies

Interventional agents	Species	Results	References
CeNP@MnMoS_4_	PC12 cells	This agent curtailed oxidative stress and stimulated neurite outgrowth, while hindering β-amyloid that had been instigated by Cu^2+^	[112]
MoS_2_	SH-SY5Y cells (human neuroblastoma cells)	The nanoparticle obstructed Ca^2+^ channel development and β-amyloid to minimize oxidative stress	[102]
Mo polyoxometalate complexes	PC12 cells	Both Cu^2+^ and Zn^2+^ were effective in instigating β-amyloid (Aβ_40_) accumulation, which was hindered following treatment with these agents	[113]
		Also, apoptosis, mitochondrial membrane potential depolarization and oxidative stress were impaired by Mo polyoxometalate complexes	
MoS_2_/AuNR	SH-SY5Y cells (neuroblastoma)	This nanocomposite, beside disaggregating and hindering β-amyloid (Aβ_42_) fibrils, also alleviated ROS triggered by β-amyloid	[114]
MoS_2_	–	This agent regulated β-amyloid (Aβ_33–42_) by inhibiting its agglomeration	[115]
MoS_2_-CoDOTA	PC12 cells (rats)	The nanoparticle crossed the BBB to significantly hinder and degrade β-amyloid fibrillations, along with curtailing cytotoxicity instigated by β-amyloid	[116]
MoS_2_ QDs	–	The agent, as an immunosensor, discerned β-amyloid levels in a precise manner	[117]
FeOOH/Mo:BiVO_4_	Human tau_441_	Doped Mo ions and FeOOH integrated into BiVO_4_ photoelectrode augmented the photocurrent impulses of DAB to diagnose t-tau proteins	[118]

Mo, Molybdenum; DAB-3,3-Diaminobenzidine; MoS_2_, Molybdenum disulfide; AuNR, gold nanorods; QDs, Quantum dots; PC12, pheochromocytoma cells; BiVO_4_, Bismuth vanadate; FeOOH, Iron oxyhydroxide; CoDOTA, Cobalt 1,4,7,10-Tetraazacyclododecane-1,4,7,10-tetraacetic acid; CeNP@MnMoS_4_–; t-tau, total tau

In summary, Mo has an immense potential to serve as an interventional agent against AD. However, further thorough studies are required, most especially to ascertain the appropriate concentration necessary to achieve the desired outcome in AD.

### Iodine: a familiar, but an unchartered biometal in AD

Studies have used iodine-containing agents as diagnostics in AD [119, 120]. Notably, a fabricated (123) I-ABC577 agent, (a subsidiary of radio-iodinated imidazopyridine) was tested in animal and human models of AD and ascertained to be a possible single photon emission computed imaging biomarker for the diagnosis of β-amyloid [121]. The investigation into the catabolism of β-amyloid (Aβ_40_ and Aβ_42_) in mice (APPswe/PS1dE9) and humans showed peripheral organs, such as kidney, skin, liver and gastrointestinal tract to be prominent in clearing β-amyloid, consequently curtailing its load and mitigating neuroinflammation (via decreased TNF-α, IL-6, IL-1 cytokines, microgliosis and astrocytosis), tau phosphorylation (via minimized pS396 and pS199) and neuronal degeneration (via augmented synaptophysin, PSD93/95 and synapsin-1 levels) in the hippocampus and neocortex [122]. This study opens the avenue of targeting β-amyloid clearance in the periphery as a viable treatment for AD, as developing interventional agents that accelerates the catabolic activities of these peripheral organs could be beneficial for the disease. β-amyloid and oxidative stress appear to go hand-in-hand as evidenced from previous reports [1, 123], in that, decreased β-amyloid curtails ROS and vice-versa. Thus, using the study [122] as a rationale, it is possible that decreased oxidative stress in the periphery may potentially abate β-amyloid in the peripheral region, which in turn could minimize cumulated β-amyloid in the brain. We refer to a recent study that established the association between iodine and oxidative stress occurring in the periphery [69]. Dietary, but not supplemental, iodine was demonstrated to regulate plasma levels of both glutathione peroxidase and triiodothyronine in Rex rabbits. In particular, glutathione peroxidase was elevated, leading to the suppressed formation of H_2_O_2_ [69]. Exacerbated level of H_2_O_2_ causes oxidative stress damage, having been reported and reviewed extensively [124, 125]. In pregnant hypertensive women lacking iodine, excessive degrees of oxidative stress (as measured by thiobarbituric acid reactive substance, TBARS) along with diminished activities of catalase and SOD were noted [126]. Moreover, 196 children (between the ages of 9 and 16) with moderate iodine insufficiency showed exacerbated oxidative stress (as confirmed by higher total oxidant status and lower total antioxidant status) [127]. One of the intricacies surrounding AD pathogenesis has to do with materialization of symptoms years after associated possible risk factors have been triggered. As such, the early dysregulation of oxidative stress in the children [127] and pregnant women (especially if young) [126] might have potentially stimulated the aberrant formation of β-amyloid in these individuals, which in turn would have already placed them in a precarious position of likely developing a neurodegenerative disease like AD in later years (say between 20 and 30 years from time of study conclusion). Although this is from a hypothetical viewpoint, continual observation of the health status of these individuals could be paramount. Additionally, animal models investigating the long-term effect of iodine deficiency in AD development could be significant, as it would either refute or substantiate our hypothesis. In the event of substantiating, it could open avenues for both understanding and tackling the disease. Future studies in this area could focus on (1) ascertaining which of the two (either iodine supplements or dietary iodine) has beneficial effects in combating oxidative stress in AD, and (2) establishing the optimal daily iodine intake (either dietary or supplements).

Iodine may be related to cognitive function. A post-mortem study was conducted to assess iodine levels in various sections of the brain. Although iodine was observed to be generally low and unevenly dispersed, its highest levels were discerned in the putamen, frontal cortex, and caudate nucleus [128]. Noteworthy is that these brain regions have been intensely investigated and implicated in cognitive functions [129–132]. It is also interesting to note that the brain samples used for the study were from humans who had no history of either psychiatric or neurological conditions. Based on this result, does it mean that iodine levels increase with age? Despite the absence of specific evidence addressing the above quandary, data from the National Health and Nutrition Examination Survey (NHANES), which evaluated the iodine status of the US population between 2007 and 2008 agree with the statement that iodine increases with age. In that study, the median urine iodine concentration of those between 50 and 59 years was 149 μg while that of 60–69 years and ≥ 70 years were 165 μg and 187 μg respectively. Interestingly, the NHANES study conducted between 2005 and 2006 showed similar outcome [133]. As there is no other available literature data demonstrating the normal iodine levels in the human brain by age group, it is difficult to draw any meaningful conclusion from the study conducted by Pinto et al. [128]. In this regard, future studies focusing on this area is paramount as results from investigations could determine whether iodine may be a prospective interventional agent for AD.

## Conclusion and future directions

Mo and iodine are closely related to AD. These less investigated trace biometals have positive impacts—for instance, modulation of inflammation, oxidative stress, and β-amyloid proteins. More so, they can detect β-amyloid levels in the brain. With the aim of further exploring the applicability of these biometals not just as interventional agents, but also as diagnostic tools for AD, future studies could attempt to:

1. Ascertain the normal level of iodine in the human brain.
2. Establish the probable mechanism involved in iodine’s role in brain function.
3. Investigate the therapeutic effects of iodine in AD mice models and extend to patients.
4. Establish the functional role of Mo in AD, and strategies in utilizing it to either remedy or counteract the disease, thereby preventing disease development and progression.
5. Determine the beneficial effects of dietary and supplementary iodine in repudiating oxidative stress.

Other future studies could also attempt to ascertain the specific contribution of Zn/Cu in AD and their optimal concentration needed to improve synaptic plasticity or decrease accumulated β-amyloid.

## Data Availability

Not applicable.

## Abbreviations

AD: Alzheimer disease
ADCS-ADL: Alzheimer Disease Co-operative Study Activities of Daily Living Inventory
AMPA: α-Amino-3-hydroxyl-5-methyl-4-isoxazolepropionate-acid
APP: Amyloid precursor protein
APP/PS1: Amyloid precursor protein/presenilin 1
β-amyloid: Beta-amyloid
BACE-1: Beta-site amyloid precursor protein cleaving enzyme-1
BBB: Blood–brain barrier
BDNF: Brain-derived neurotrophic factor
CAT: Catalase
CNS: Central nervous system
COX: Cyclooxygenase
CSF: Cerebrospinal fluid
Cu: Copper
EAD: Early-stage Alzheimer’s disease
GluN2A: Glutamate (NMDA) receptor subunit epsilon-2
GPx: Glutathione peroxidase-1
H_2_O_2_: Hydrogen peroxide
IL: Interleukin
LAD: Late-stage Alzheimer’s disease
LTD: Long-term depression
LTP: Long-term potentiation
MCI: Mild cognitive impairment
MMP: Matrix metalloproteinases
Mo: Molybdenum
MoS_2_: Molybdenum disulphide
MT: Metallothionein
NF*-*κB: Nuclear factor kappa-light-chain-enhancer of activated B cells
NFT: Neurofibrillary tangles
NHANES: National Health and Nutrition Examination Survey
NMDA: *N*-Methyl-d-aspartate
NO: Nitric oxide
PCAD: Preclinical Alzheimer’s disease
PIN-1: Peptidyl-prolyl cis–trans isomerase NIMA-interacting 1
ROCK: Rho-associated protein kinase
ROS: Reactive oxygen species
SLC: Solute carrier
SOD: Superoxide dismutase
TBARS: Thiobarbituric acid reactive substance
TNF-α: Tumour necrosis factor-alpha
TrkB: Tyrosine kinase B
ZiP: Zinc-regulated and iron-regulated transporter proteins
ZIP: Zrt- and Irt-like protein
Zn: Zinc
ZnSO_4_: Zinc sulphate
ZnT: Zinc transporter
